# Current trends in surgical management of myopia

**Published:** 2019-05-13

**Authors:** Javed Hussain Farooqui, Manisha Acharya, Mitali Kekan

**Affiliations:** 1 Clinical Fellow: Department of Cornea and Refractive Surgery, Dr Shroff's Charity Eye Hospital, New Delhi, India.; 2 Senior Consultant: Department of Cornea and Refractive Surgery, Medical Director - Eye Bank, Dr. Shroff's Charity Eye Hospital, New Delhi, India; 3 Clinical Fellow: Department of Cornea and Refractive Surgery. Dr. Shroff's Charity Eye Hospital, New Delhi, India.

**We describe various surgical options for correction of myopia, the advantages and disadvantages of the procedures, current practices and emerging trends.**

**Figure 1:**
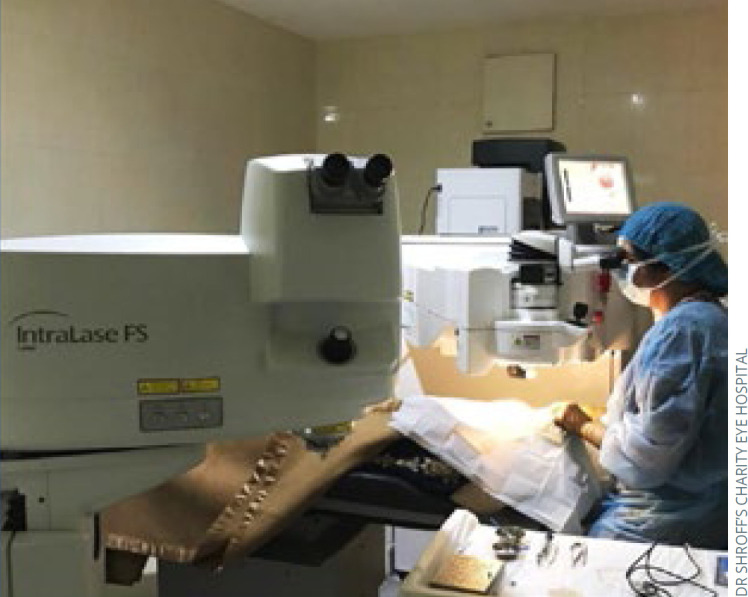
Surgeon performing LASIK surgery.

Over the last few decades, refractive surgery for treatment of myopia has gained popularity as both a cosmetic procedure to avoid spectacles and as a means of complying with occupational vision standards. Refractive surgery procedures include:

- Incisional refractive surgery
- Excimer laser refractive surgery and
- Intraocular surgery

## Incisional refractive surgery

The first report of using an incision to alter the shape of the human cornea was in the 19^th^ century, when Schiotz^1^ used a limbal relaxing incision in a patient who underwent cataract surgery. In the 1980s Radial Keratotomy (RK) was used to treat thousands of patients with myopia with good predictability; however complications including infection, weakening of the cornea and night vision problems has made RK a more or less obsolete procedure for myopia management.

## Excimer laser refractive surgery

- Photorefractive keratectomy (PRK)
- Laser subepithelial keratectomy (LASEK) and
- Epithelial laser in situ keratomileusis (epi-LASIK, referred to as LASIK)

In PRK, the epithelium is removed either mechanically by scraping it with a blade or chemically by using a diluted solution of ethanol. In the latter approach the epithelial sheet is not repositioned after laser ablation.

In LASEK the epithelial flap is repositioned gently over the ablated tissue.

**Figure 2A & B:**
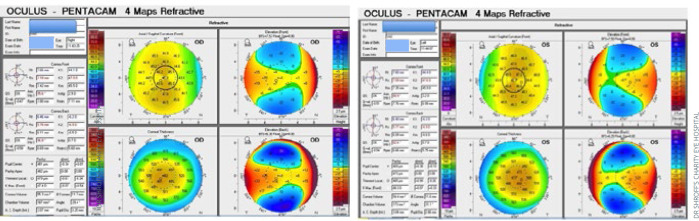
Pentacam image of right and left eye, performed in the pre-operative workup period

Comparative studies of surface ablation techniques (PRK versus LASEK) have shown similar refractive outcomes. These procedures work best for myopia up to 6.0 D (dioptres). Laser surface ablation is a better option than LASIK in patients with epithelial irregularities, dry eye syndrome, thin corneas, possible risk of post LASIK flap dislocation and possible risk of keratectasia.

LASIK is currently the most popular surgical option for myopia correction. It is superior to PRK in terms of patient comfort, visual stabilisations and stromal haze formation. It was first popularised by Pallikaris^2^ and Buratto^3^ as a technique of laser ablation of the corneal stroma, which involved the creation of a flap of anterior stroma including Bowman's and epithelium with the aid of a microkeratome. It can be used to treat up to 15.0 D of myopia, however due to risk of long-term ectasia, the recommendation has been revised to a maximum of minus 10.0 D (Figure 1).

As with any other surgery, LASIK also has its own share of complications; these include free flaps, buttonhole flaps, irregular flaps and post LASIK traumatic flap displacement; epithelial ingrowth and dry eye syndrome also need to be kept in mind.

**Figure 3:**
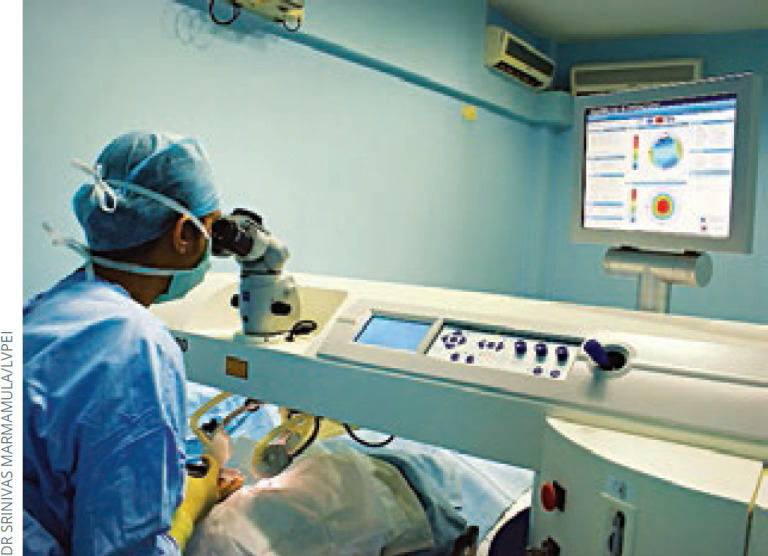
Refractive lens exchange (RLE)

**Figure 4:**
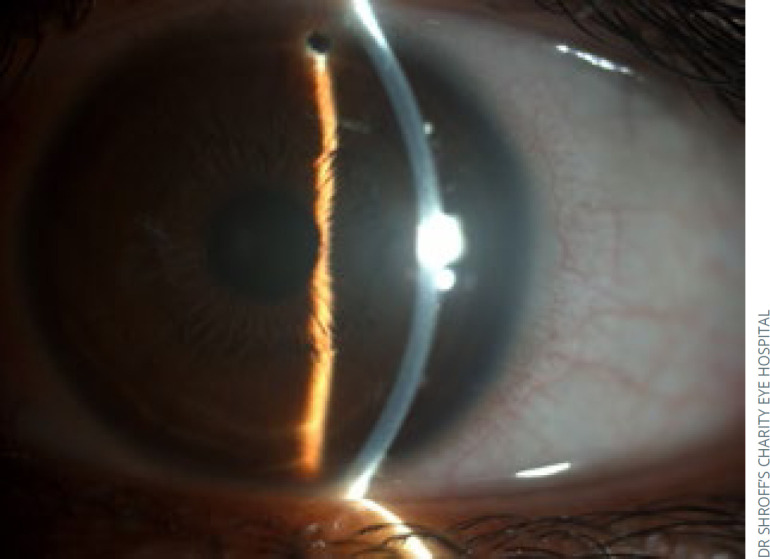
Implantation of a Phakic Intraocular Lens (pIOL)

Recently, Wavefront-guided LASIK is being used to preserve the asphericity of the cornea, thus inducing less spherical aberration compared with standard LASIK (Figure 2A and B).

## Intraocular surgery

For very high degrees of myopia (more than minus 10.0 D), LASIK is unpredictable and runs a risk of regression and ectasia. Refractive lens exchange (RLE), also known as clear lens extraction, was first described by Fukala in 1890, as one of the options to treat high myopia (Figure 3).^4^ The availability of a wide range of lens powers both for sphere and cylinder have made this approach more attractive when LASIK is contraindicated. This procedure is best suited for treating high myopia up to myopia −23 D, or in other myopes where optical correction or refractive surgery is contraindicated. Even though good refractive outcomes are reported, high myopes run a risk of retinal detachment after surgery, a complication that the patients should be counseled about. Implantation of a pIOL can also be considered (Figure 4).

## Recent advancements

These days, Femtosecond lasers^5^ (IntraLase, Irvine, California, USA) have been reported to create more accurate and thinner flaps resulting in more predictable results. The flap dimension can be adjusted based on the needs of the patient and the type of excimer laser used. This also aids in faster recovery, and reduces the risk of further corneal problems.

The most recent addition to this list is that of Small incision lenticule extraction (ReLEx® SMILE) which is a minimally invasive procedure, and combines the advantage of both PRK and LASIK that is flapless and fast recovery.^6^ During this procedure, an intrastromal lenticule is created, and is removed from a small tunnel incision. Since 2011, when the procedure started in Europe, China and India, it has proved to be ideal.

Surgical options for refractive errors have come a long way since 1990. In a field like ophthalmology, which is dependent on technology, we can only expect that treatment options will become more sophisticated and patient-friendly in the coming years.
